# Synergistic inhibition of gastric cancer cell proliferation by concanavalin A and silibinin via attenuation of the JAK/STAT3 signaling pathway and molecular docking analysis

**DOI:** 10.1186/s41065-025-00438-z

**Published:** 2025-05-11

**Authors:** Gaoyan Hua, Lisha Zhao, Xianjing Zeng, Liang Luo

**Affiliations:** 1 Department of Oncology, The People’s Hospital of Chizhou, Chizhou, Anhui 247001 China; 2 Department of Gastroenterology, Tianyou Hospital Affiliated to Wuhan University of Science and Technology, Wuhan, Hubei 430000 China; 3 General Practice Medicine, Affiliated Hospital of Jinggangshan University, Ji ’an, Jiangxi 343000 China; 4 Department of Oncology, Affiliated Hospital of Jinggangshan University, Ji ’an, Jiangxi 343000 China

**Keywords:** Concanavalin A, Silibinin, Cell proliferation, JAK/STAT3, Gastric cancer

## Abstract

**Background:**

In the current period of pharmaceutical discovery, herbal remedies have shown to be an unmatched supply of anticancer medications. Plants and their derivatives, through analogues, play a vital role in cancer treatment.

**Objectives:**

The current investigation assessed the effectiveness of inhibiting the growth of gastric cancer cells in AGS cells by blocking the JAK/ STAT3 signalling pathways using the natural medicines Concanavalin A (Con-A) and silibinin (SB).

**Materials and methods:**

After being exposed to various doses of concanavalin A, and silibinin (Con-A + SB) for 24 h (0- 60 µM), the cells were evaluated for multiple studies. The MTT assay was used to examine the combination of Con-A + SB-induced cytotoxicity. To evaluate ROS, DCFH-DA staining was utilized. Dual (AO/EtBr) staining was performed to examine apoptotic modifications, and MMP levels in AGS cells were examined using the appropriate fluorescence staining assays. By using flow cytometry and western blotting, cell cycle, and apoptosis were assessed.

**Results:**

The relative cytotoxicity of Con-A and SB was found to be approximately 19.6 μM and 16.78 μM, (*p* < 0.05) correspondingly, according to the findings. After a 24-h incubation period, the combination of Con-A and SB generates significant cytotoxicity in AGS cells, with an IC_50_ of 10.37 μM (*p* < 0.01). Furthermore, AGS cells treated with Con-A and SB concurrently showed increased apoptotic signals, exhibited by Bax overexpression, Bcl-2 downregulation, and Caspase-3 activation, as well as considerable ROS generation.

**Conclusion:**

Therefore, the combination usage of Con-A + SB has the potential to serve as a chemotherapeutic agent since it prevents the synthesis of JAK/STAT3 intermediated control of proliferation and cell cycle-regulating proteins.

**Supplementary Information:**

The online version contains supplementary material available at 10.1186/s41065-025-00438-z.

## Background

Carcinoma was formerly thought to be intractable, but due to scientific advancements, there is now a lot of hope for treating those who have been diagnosed with the disease in its early stages. According to Cancer Statistics, nearly 2.0 million instances of cancer and 611,720 mortality are projected in the United States in 2024, emphasizing the rising cancer impact [1]. Gastric cancer (GC) is an epithelial cancer that develops in the gastrointestinal tract. It ranks fifth (5.6%) in incidence and fourth (7.7%) in mortality globally, with an anticipated 970,000 new cases and more than 720,000 fatalities in 2024 [2]. Because of the lack of early clinical symptoms, more than 70% of GC patients are detected at advanced stages, causing in a dismal prognosis, low survival rates, and restricted therapeutic choices [3]. As noted in the NIH assessment, despite breakthroughs in surgery, chemotherapy, immunotherapy, and targeted therapies, there is an urgent need to identify novel, effective and imaginative therapies to improve patients'prognosis and overall quality of life [4].

To this point, a considerable amount of research has been presented about the factors that contribute to GC development. It appears that complicated molecular pathways constitute especially significant factors in GC malignancy [5, 6]. The discovery of these mechanisms may pave the way for successful GC treatment. Experiments in this field have shown that many biochemical pathways and their mediators play a role in GC formation [7, 8]. Multiple recent investigations have shown that signal transducer and activator of transcription (STAT3) is active by default in a variety of malignant tumors and tumor cell lines, including breast cancer, prostate cancer, pancreatic adenocarcinoma, colon carcinoma, and GC [9, 10]. The activated STAT3 is frequently linked with tumor invasion, metastasis, and prognosis by increasing cancer cell proliferation, survival, and angiogenesis [11]. Additionally, inhibiting STAT3 activity can limit cancer cell proliferation and invasion while inducing apoptosis [12]. pSTAT3 is an essential component of the JAK/STAT pathway. As a consequence, the JAK-STAT pathway is regarded to be a promising target for cutting-edge cancer therapy. Therefore, to be among the primary areas of research for the development of new anticancer drugs is the inhibition of the JAK/STAT3 signaling pathway.

The development of efficient and specific inhibitors of small molecules in the STAT3 signaling system has proven challenging and contentious. Identifying new tiny molecules in substances made from plants is one method for developing novel pharmaceuticals [13]. Several natural chemicals exhibit significant antimicrobial and anticancer properties, which ultimately contributed toward their potential development as cancer therapies [14, 15]. A disease can be better understood as a network of interlinked cellular processes that are highly susceptible to the simultaneous action of several drugs. This allows for more in-depth investigations of drugs combination [16]. As two drugs that cause an identical broad therapeutic response are combined, they can provide the same result in varying magnitudes as compared to the total of the effects of separate agents. This impact might be more, equal, or less than the corresponding summation, indicating synergy, antagonistic activity [17]. Therefore, when two or more medications function synergistically, lesser dosages of each can accomplish the intended objective, including cancer cell killing, while reducing the undesirable effects connected with greater doses. Because of the aforementioned benefits, medication combinations are an essential therapeutic method that has become widely employed in the treatment of several diseases, including infectious disorders and various forms of malignancy. There have been several studies reported on the usefulness of bioactive naturally occurring substances in treating stomach cancer [18]. However, until recently, research has only looked at natural resources as independent therapies; not any studies have looked at the combined effects of utilizing natural products. Thus, the current study investigated the synergistic impact of combining natural compounds concanavalin A (Con-A) and silibinin (SB).

Concanavalin A (Con-A) is a long-studied indicative legume lectin that apparently broadens human malignancies by focusing on programmed cell death. Apoptosis and autophagy are progressive discussed interprets that involve preserving homeostasis and eradicating harmful cells. Earlier research demonstrated that Con-A caused apoptosis in human melanoma cells and cervical cancer. As a result, Con-A carries special apoptosis- and autophagy-inducing features [19, 20]. Silibinin is a flavonone produced from the milk thistle (Silybummarianum L.). This is prescribed to treat liver disorders and intoxication. Recently, silibinin has been investigated tried in treatments for cancer and has showed encouraging effects against breast, prostate, skin, lung, colon, lung, bladder, and ovarian malignancies [21, 22].

**Scheme 1:**
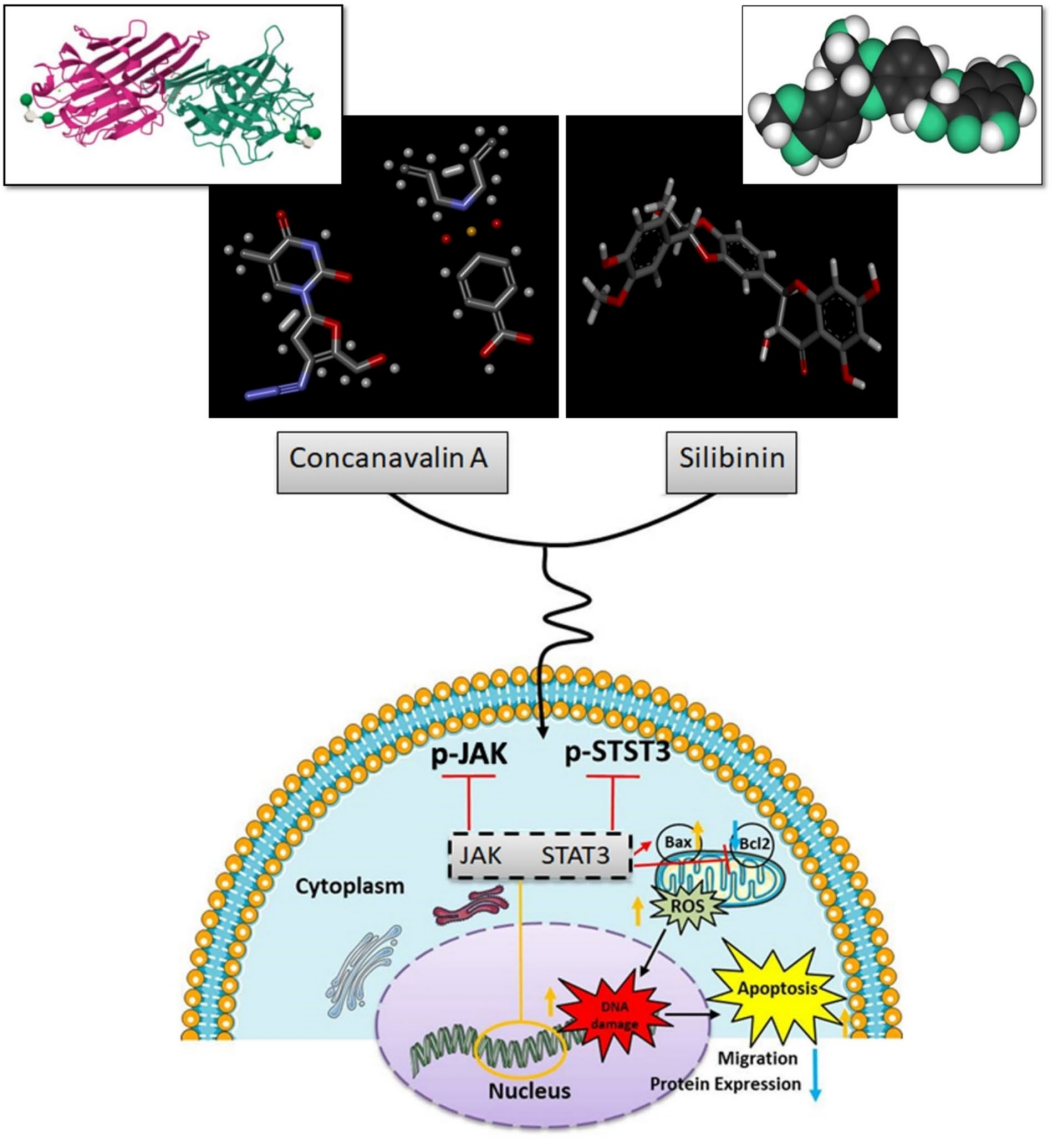
The present investigation employed Con-A and SB in combination to investigate the possibility of combinatorial chemopreventive properties towards gastric cancer cells. The current investigation concentrated on the molecular mechanisms associated in the JAK/STAT3 pathways, which might suggest repression in the initial stages of gastric cancer progression

As a result, the combination of Con-A and SB has the potential to suppress cancer cell development. However, no empirical data suggests that combining Con-A and SB has a therapeutic impact on gastric cancer, especially with target-specific mechanisms. The present investigation employed Con-A and SB in combination to investigate the possibility of combinatorial chemopreventive properties towards gastric cancer cells. The current investigation concentrated on the molecular mechanisms associated in the JAK/STAT3 pathways, which might suggest repression in the initial stages of gastric cancer progression (Scheme 1).

## Material and methods

### Sourcing chemical

Concanavalin A and Silibinin, fetal bovine serum (FBS), MTT (3-(4,5-dimethylthiazole-2-yl)−2,5 diphenyl tetrazolium bromide), and powdered Dulbecco's modified Eagle's medium) were acquired from Chemical Co. (St. Louis, MO, USA). Primary antibodies against STAT3, JAK1, Cyclin D and Cyclin E1 were obtained from Signalway Antibody Co. (College Park, MD, USA). In contrast, antibodies against β-actin, Bcl-2, Bax, Cytochrome and caspase 3 were sourced from Santa Cruz Biotechnology (Dallas, TX, USA). Signalway Antibody Co. (College Park, MD, USA) produced secondary anti-mouse (Catalog No: L3032) and anti-rabbit IgG-HRP (Catalog No: Cat. No. L3012). Gibco-BRL Co. (Gaithersburg, MD, USA) provided all of the laboratory media along with growth additives. The additional solvents and chemicals all met the scientific standard and were bought through Merck. The dissolved in DMSO, Concanavalin A and Silibinin were maintained at 4ºC.

### Molecular docking analysis

The 3D structures of Con-A and SB were retrieved from the PubChem-NCBI database, while the target proteins JAK and STAT3 were obtained from the RCSB Protein Data Bank. The protein and ligand files were prepared by converting them into pdbqt format using Autodock Tools (v1.5.7 rc1) provided by the Scripps Research Institute. A docking grid was set up, and molecular docking simulations were conducted using Autodock Vina (v1.5.6, available at https://vina.scripps.edu). The docking results were further analysed using BIOVIA Discovery Studio Visualizer software to identify interactions.

### Stability analysis of Con A and SB

Concanavalin A and Silibinin (SB) were dissolved separately in aqueous solution (PBS, pH 7.4) and DMSO. Final doses of Con A: 50 µg/mL and SB: 100 µM. To prevent freeze–thaw cycles, mixtures were filtered (0.22 μm) and divided into aliquots. Samples were kept at 37 °C in the dark to simulate cell culture conditions. Samples were collected at 0, 6, 12, 24, and 48 h. Stability was determined by measuring UV–Vis absorbance spectra using a spectrophotometer (range: 200–600 nm). The stability percentage (%) was computed through contrasting the absorbance at each time point to the initial absorbance (t = 0), given as:

$$\text{Stability }(\text{\%})= (\text{Abst}/\text{Abs}0) \times 100$$

### Acquisition and sustaining of the cell line

A human GC cell line AGS, was procured from the Chinese Academy of Sciences'Type Culture Collection located in Shanghai, China. They were cultivated in Dulbecco's modified Eagle's medium, which was enriched with ten percent (v/v) fetal bovine serum and 1% antibiotics. The culture was maintained at 37˚C in a 15% CO_2_ humidified environment. Complete media was used to suspend the loosen cells, and regular reseeding was done. For the MTT tests and the cell model used in later research, AGS cells were utilized.

### Assessment for cell viability

Using the MTT test, the impact of Con-A and SB prevention on AGS cell growth was ascertained. The cells were cultivated on 96-well tissue cultivation plates at an average density of 2500 individuals per well, and they were kept in an incubator with humidification with 5% CO_2_ at 37 ºC. Following the cells'50% confluence, they were treated with Con-A and SB before being cultured for 48 h. Following the removal of the above medium, every well was filled with 0.5 mg/ml of MTT solution (PBS and medium), and the mixture underwent incubation for 4 h. After removing the medium, 100 µl of DMSO and 25 µl of Sorensen's glycine buffer were used for dissolving the blue formazan crystals. In a microplate reader, the intensity of absorption was measured at 570 nm. Every experiment was conducted three times, and the outcomes were presented as means ± SEM.

### Assessment of Con-A and SB combination index (CI)

Compusyn Software (ComboSyn Inc., Paramus, NJ, USA) was utilized [20] to assess the anticancer effect of Con-A and SB by calculating their combination index (CI). Comparative cell viability (as a percentage of control) was computed following the analysis of MTT assay data. Next, the Chou-Talalay technique, [23] which offers a quantitative understanding of synergism and antagonistic relationships, was used to evaluate CI.

$$\text{CI}= \frac{{\left(D\right)}_{c}}{{\left({D}_{x}\right)}_{c}}+ \frac{{\left(D\right)}_{s}}{{\left({D}_{x}\right)}_{s}}= \frac{{\left(D\right)}_{c}}{{\left({D}_{m}\right)}_{c}[\frac{\frac{{f}_{a}}{1-{f}_{a}}}{1}{/}^{m2}}+\frac{{\left(D\right)}_{s}}{{\left({D}_{m}\right)}_{s}\frac{\left[\frac{fa}{\left(1-{f}_{a}\right)m}\right]{/}_{m2}}{1}}$$

While the coefficients (D)s and (D)c are the quantities of Ds and Dc in the SB‑ Con-A combination at x% inhibition setting, the denominators (Dx)s and (Dx)c are the concentration in order of single (Dx)s (S, silibinin) and (Dx)c (C, Concanavalin A) that causes x% repression. The CI < 1, = 1, > 1 reflects synergism, combined effect, and antagonistic effects in drug combinations. The meridian-effect equation of Chou et al. [24], was used to determine the (Dx)s and (Dx)c.

$${D}_{x}=\left({D}_{m}\right){\left[{f}_{a}/\left(1-{f}_{a}\right)\right]}^{1/m}$$

While Dm = 10-(Y-intercept)/m is the percentage impacted by the therapeutic dose D, and m is the gradient of the median-effect plot. Dx is the median-effect dose determined using anti-log of the X-intercept of the median-effect plot, X-log (D) vs.

### Evaluation for colony formation

The cells were cultivated for one night after plating at a density of 1,000 cells per well in 60-mm plates. Subsequently, Con-A, SB and combination of Con-A and SB were applied to the cells for 72 h. After drug treatment, the cells were incubated in drug-free medium for 14 days to allow colony formation. Subsequently, the colonies were stained with methylene blue, imaged, and counted.

### Determination of Mitochondrial Membrane Potential (Δψm)

Using the mitochondrial tracking fluorescent DiOC6(3), flow cytometry was used to quantify the ΔΨm levels, as earlier reported [25]. For the specified amount of time, AGS cells were seeded onto a culture dish and subjected to Con-A and SB. The samples were analyzed using the BD FASCanto Flow Cytometer (excitation at 484 nm) after being stained with 40 nM DiOC6 (3) for 20 min at 37 °C in the dark. For every sample, the number of cells was at least 10,000. Using CellQuest software, the flow cytometry data were examined and converted to mean fluorescence intensity (MFI). The findings of three separate assessments were represented in the data.

### DAPI staining for morphological assessment of nuclei within cells

Nuclear shrinkage and disintegration were detected in the cells using the fluorescent substance 40,6-diamidino-2-phenylindole (DAPI), which was used to evaluate apoptosis following drug therapy. In summary, 12 well plates were loaded with cells, and they received treatments with Con-A, SB, and the combination thereof. Following the incubation stage, the cells were fixed for eight minutes at room temperature using 250 mL of paraformaldehyde (4%) solution, and then they were rinsed multiple times in phosphate-buffered saline (PBS). Following a 10-min permeabilization with 0.1% Triton-X 100, the fixed cells were again cleaned with PBS and allowed to sit for five minutes. Lastly, cells had been stained at ambient temperature in a dark environment using a DAPI solution (2 mg/mL) (Hi-media). Fluorescence microscopy was used to analyze the cells'nuclear structure.

### DNA fragmentation measurement using electrophoresis

After being separated into drug-treated cells (1 × 10^6^) and cells that were untreated (control), the cells were lysed in a solution containing proteinase K (1 mg/mL, Serva, Germany) and 10 mmol/l TRIS, 10 mmol/L EDTA, and 0.5% Triton X-100. After one hour of incubation at 37 ºC, samples underwent heating for ten minutes at 70 ºC. After the lysate had been included, RNA-ase (200 g/mL, Serva) was added, and the mixture was once again incubated for one hour at 37 ºC. The samples were run through 1.5% (w/v) agarose gels (Sigma) supplemented with 1 g/mL of ethidium bromide at 40 V. DNA ladders, or segregated DNA pieces, were visible under a UV transilluminator. The fragments of DNA were sized by contrasting them to the Superladders-Mid2 200 bp ladder, which is a database of DNA molecular weight indicators.

### Comet assay

Con-A, SB, and a combo of Con-A + SB were all applied to the cells for 48 h. Cells have been obtained and washed on three separate occasions in PBS following incubation. Following processing for the Comet test, the cell pellet was suspended in 100 ml of PBS. Electrophoresis on a single cell alkaline gel was carried out as previously. The following day, an image analysis system connected to an Olympus fluorescence microscope was used for the slides'analysis and scoring. As the primary metric for evaluating cell DNA damage, tail length—or the movement of DNA from the nucleus—was evaluated.

### Apoptosis determination

The Annexin V-FITC/PI Apoptosis Measurement Kit found the apoptosis rate in accordance with the manufacturer's instructions. For twenty-four hours, AGS cells were grown with Con-A, SB, either alone or in combination of Con-A + SB. Following harvesting, cells were resuspended in 0.5 mL of binding solution containing 40 ng/sample of PI and Annexin-V (1:50) and incubated for 30 min at 37 °C under dark conditions. BD FASCanto flow cytometer and CellQuest software were used to examine the samples. As stated in reference [25], the apoptosis rate (%) is calculated as follows: (apoptotic cell count/total cell count observed) × 100%.

### Examining the cell cycle with flow cytometry

One non-specific DNA probe that creates a fluorescent compound in DNA is propidium iodide (PI). When exposed to UV light, PI becomes excited and releases red fluorescence. The amount of DNA is shown by the fluorescence's amplitude. By assessing the amount of cellular DNA, flow cytometry is a useful technique for determining the cell cycle phase. Following treatment, each group's cells were mounted in cold 70% ethanol per the kit's instructions, and then they were stained with 50 μg/ml PI incorporating 20 μg/ml RNase. Lastly, they underwent analysis using FlowJo software [26], and tested using flow cytometry (Mindray, China).

### Western blot

AGS cells were propagated at an average density of 5 × 10^6^ cells/well in a culture dish throughout the phase of logarithmic development, as reported before [11]. Following incubation for 24 h, cells were treated for 6–24 h with Con-A and SB. Following cell harvesting, ice-cold PBS was used twice for washing. To remove the supernatant from whole cell lylates, 1.5 mL Eppendorff tubes holding cells were spun at 110 g for 5 min. After vortexing the pellet, 100 μL of 1 × loading buffer were applied to each well. The lysates were heated to 100 °C for 20 min. Afterward, they were centrifuged at 15,000 g for 10 min in an Eppendorff, and the supernatant was obtained. To obtain the subcellular portion, the cells were suspended in 1.5 mL Eppendorff tubes containing a fivefold amount of ice-cold cell extract buffer, and the tubes were then incubated at 4 °C for 40 min. Following that, the cells were centrifuged for 10 min at 4 °C at 110 g. After centrifuging the supernatant for 15 min at 4 °C at 15,000 g, the resulting supernatant was utilized to create the cytosolic fraction. After that, the previously obtained supernatant was combined with 5 × loading buffer and heated to 100 °C for 15 min.

After that, proteins were separated using 8%–12% sodium SDS-PAGE, electrotransferred to PVDF membranes (Millipore, Billerica, MA, USA), and probed using primary antibodies designed for the proteins before being exposed to a secondary antibody conjugated with HRP. Using a PhototopeTM-HRP Detection Kit (Cell Signaling, Danvers, MA, USA) and a Kodak medical X-ray processor (Kodak, Rochester, NY, USA), protein bands was found. A particular primary antibody was used to identify the proteins of STAT3, JAK1, cyclin D1, cyclin E1, PCNA, Bcl-2, Bax and caspase-3 in a proportion of 1:1,000. Secondary antibodies conjugated with HRP have been diluted 1:2000.

### Analytical statistics

IC₅₀ values were determined using nonlinear regression analysis with GraphPad Prism 9 (GraphPad Software), data analysis and visualization. One-way ANOVA was utilized for multiple groups comparisons, and an unpaired t-test was employed for comparing the mean difference between two groups. Data were presented as Mean ± SD. All the experiments were conducted three times independently.

## Results

### Stability analysis of Con A and SB

**Fig. 1:**
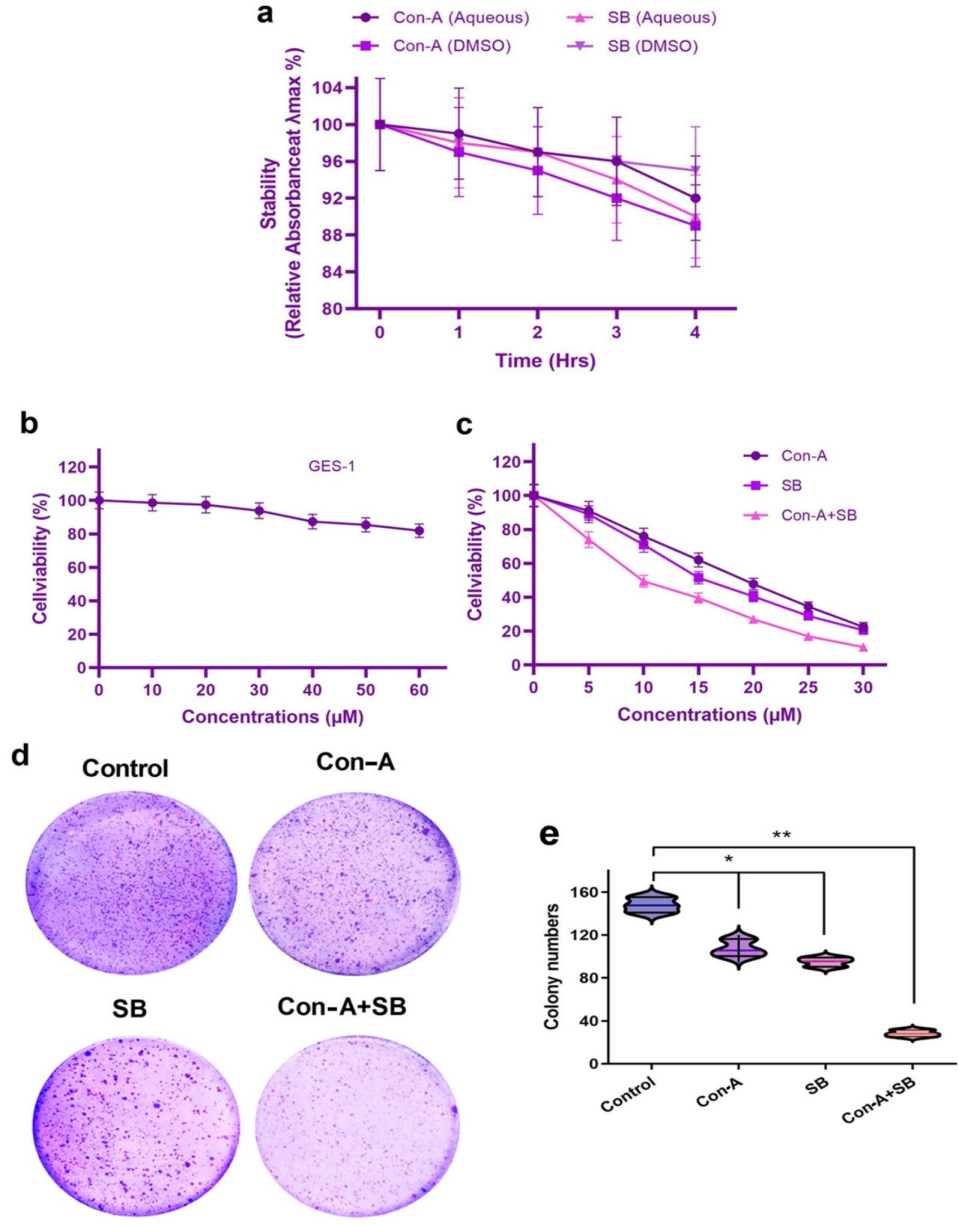
The impact of Con-A and SB combinations on AGS cell growth. **a** The different concentrations of (0–60 µM) Con-A and SB were combined to treat the human gastric epithelial cell line GES-1. **b** Con-A, SB and simultaneous administration of concanavalin A and silibinin were applied to AGS cells for 24 h at a fixed concentration (0–30 µM). The MTT assay was used to evaluate cell viability. **c**, **d** The capacity of gastric cancer cells in IC_50_ concentrations of Con-A, SB and the combination of Con-A + SB to form colonies was assessed using the colony formation test. The significance level difference comparing the individual drugs and the combined dosage indicates *p < 0.05 and **p < 0.01

Con-A and SB were tested for stability under in vitro conditions (37 °C, light-protected) (Fig. 1a). UV–Vis absorbance spectra were taken at 0, 6, 12, 24, and 48 h in both DMSO and aqueous phosphate-buffered saline (PBS) solutions. Con-A showed constant stability in aqueous buffer, with absorbance retention around 95% for up to 24 h and a little drop to around 91% at 48 h. Con A showed slightly lower stability in DMSO, with absorbance dropping to ~ 88% after 48 h. SB had good chemical stability in DMSO, sustaining more than 96% absorbance at all time points. SB stability in aqueous buffer remained high up to 24 h (~ 93%), with a little reduction to ~ 89% after 48 h. No substantial degradation, precipitation, or transformation in color was seen in any of the samples during the investigation, showing that both compounds maintained their physicochemical integrity under the circumstances used.

### Impact of Con-A and SB with varying doses affects the viability of AGS cells

Our study involves a thorough experimental examination of the anticancer effects of Con-A and SB, and their combination on AGS cell lines. The two of their combined influence on the cell proliferation capabilities were examined in order to assess the growth-inhibitory qualities of Con-A and SB (Fig. 1). Cells treated to varying doses of SB and Con-A were examined using the MTT test. The vitality of the normal cells did not alter substantially, as Fig. 1b illustrates. AGS cell line viability was significantly decreased by concurrently administering Con-A and SB in a dose-dependent manner, as demonstrated in Fig. 1c (*p* < 0.01). The IC_50_ value of Con-A, SB and combination of Con-A and SB were 19.6 μM and 16.78 μM and 10.37 µM respectively (*p* < 0.05), indicating a statistically significant difference in potency. Concanavalin A and silibinin are effective treatments for gastric cancer cells, according to these data. Based on the IC_50_ concentration further study have been carryout. Additionally, the usage of drugs combinations was found to exacerbate this impact. The treatment of Con-A, SB and simultaneous treatment of Con-A and SB related cell growth was also investigated using the crystal violet assay, which is depicted in Fig. 1d, e. The control cells have more noticeable crystal violet staining, which suggests a high degree of cell proliferation. Nonetheless, the concomitant administration of SB and Con-A significantly lowered the rate of proliferation of cells in cancer of the gastric system.

### Combination index (CI) for SB and Con-A

The CompuSyn application was used to calculate the combination index (CI). Subsequently was found that the concentrations of 10 µM, 20 µM, and 30 µM Con-A and SB concurrently lowered cellular viability in gastric cancer cell lines. Table 1 shows the CI indices for some of the combos. The presence of antagonism involving the two chemicals is indicated by a CI value greater than 1. There appears to be synergy between the two elements based on the values of CI = 1 and < 1. In AGS cells, the CI value at 5 µM Con-A and 5 µM SB is in the synergism region (CI 0.5), though the CI values at 10 µM Con-A and 15 µM SB show considerable synergism (CI 0.56, and 0.62).

**Table 1:** Combination index values for Con-A and SB (1:1)

Concentrations	CI Value	CI effect
5 µM	0.52376	Synergistic
10 µM	0.56157	Synergistic
15 µM	0.62536	Synergistic
20 µM	0.70236	Moderate Synergistic
25 µM	0.71437	Moderate Synergistic
30 µM	0.78345	Moderate Synergistic

### Assessment of nuclear morphology using DAPI Staining

**Fig. 2:**
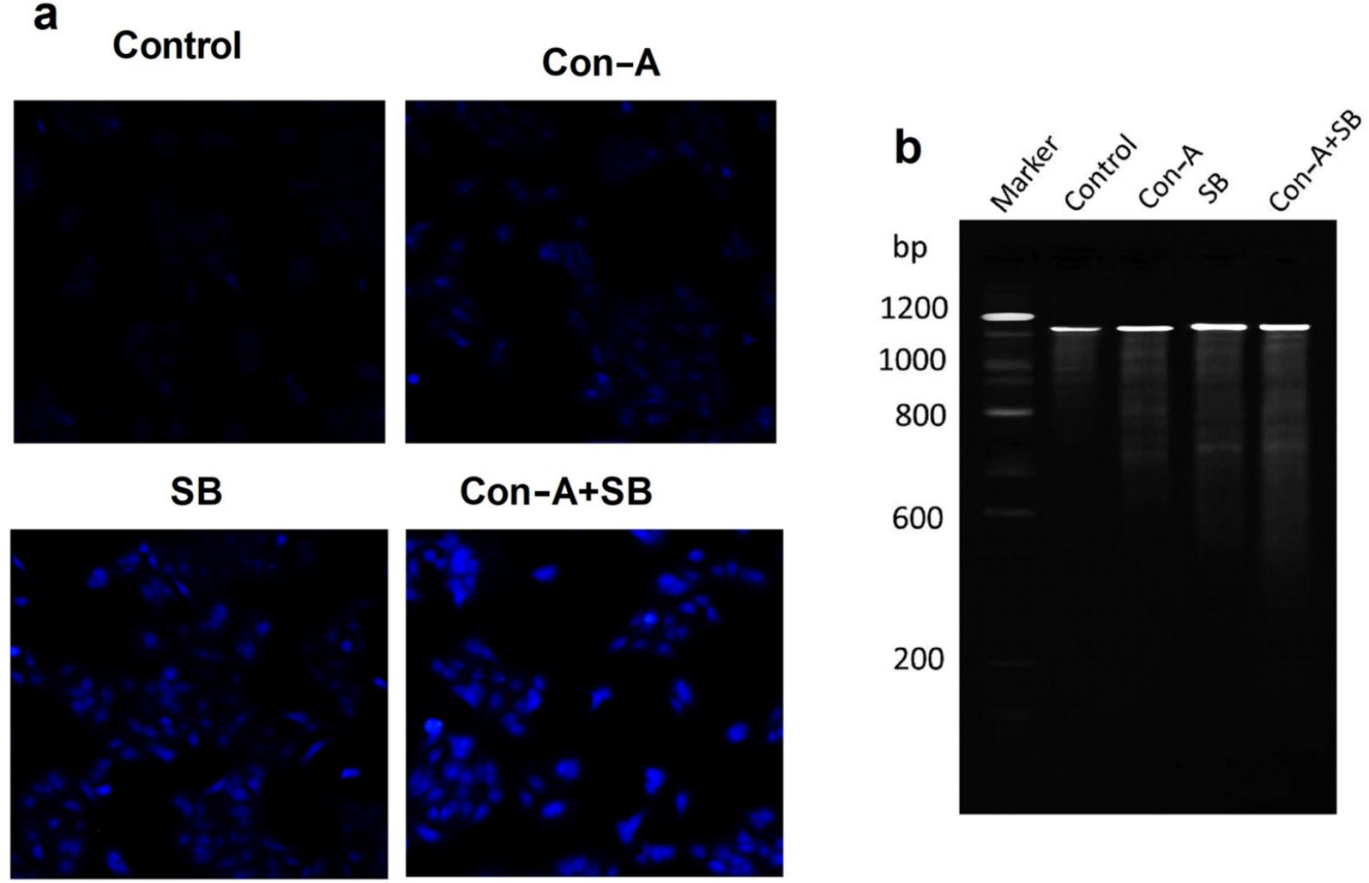
**a** AGS cells administered at IC_50_ doses with Con-A, SB and combo of Con-A and SB, alongside control nuclei stained with DAPI. **b** Detection of DNA fragmentation in AGS cells following treatment with Con-A, SB and combination of Con-A + SB. Chromosomal DNA was extracted after treatment with IC_50_ concentrations of Con-A, SB and Con-A + SB

The cells were labeled with DAPI in order to see the modifications in nuclear morphology that occurred after they were exposed to IC_50_ concentration of Con-A, SB and combo of Con-A and SB. According to these findings, AGS cells treated for 24 h with a combination of Con-A + SB showed changed nuclear architecture. The structure of the control cells was consistent or usual. On the other hand, as shown in Fig. 2a, nuclear morphology revealed an increase in nucleus condensate, nuclei blebbing, nucleus disintegration and general alterations in shape after 48 h of treatment with C_50_ concentration of Con-A, SB and Con-A + SB combination. Therefore, it has been demonstrated that these adverse outcomes have risen simultaneously with the combination treatment of Con-A and SB, as shown in Fig. 2.

### DNA disintegration

DNA fragmentation was also used to investigate the apoptosis provoked by the IC_50_ concentration of Con-A, SB and Con-A + SB combo at dose dependent manner. By using electrophoresis, disintegration in 1.5% agarose was detected. The cells given a combo of Con-A + SB showed greater degree of DNA fragmentation; the control cells, on the other hand, showed an intact nucleus without any fragmentation pattern, as seen in Fig. 2b.

### Combination of Con-A + SB increased the DNA damage in AGS cells

**Fig. 3:**
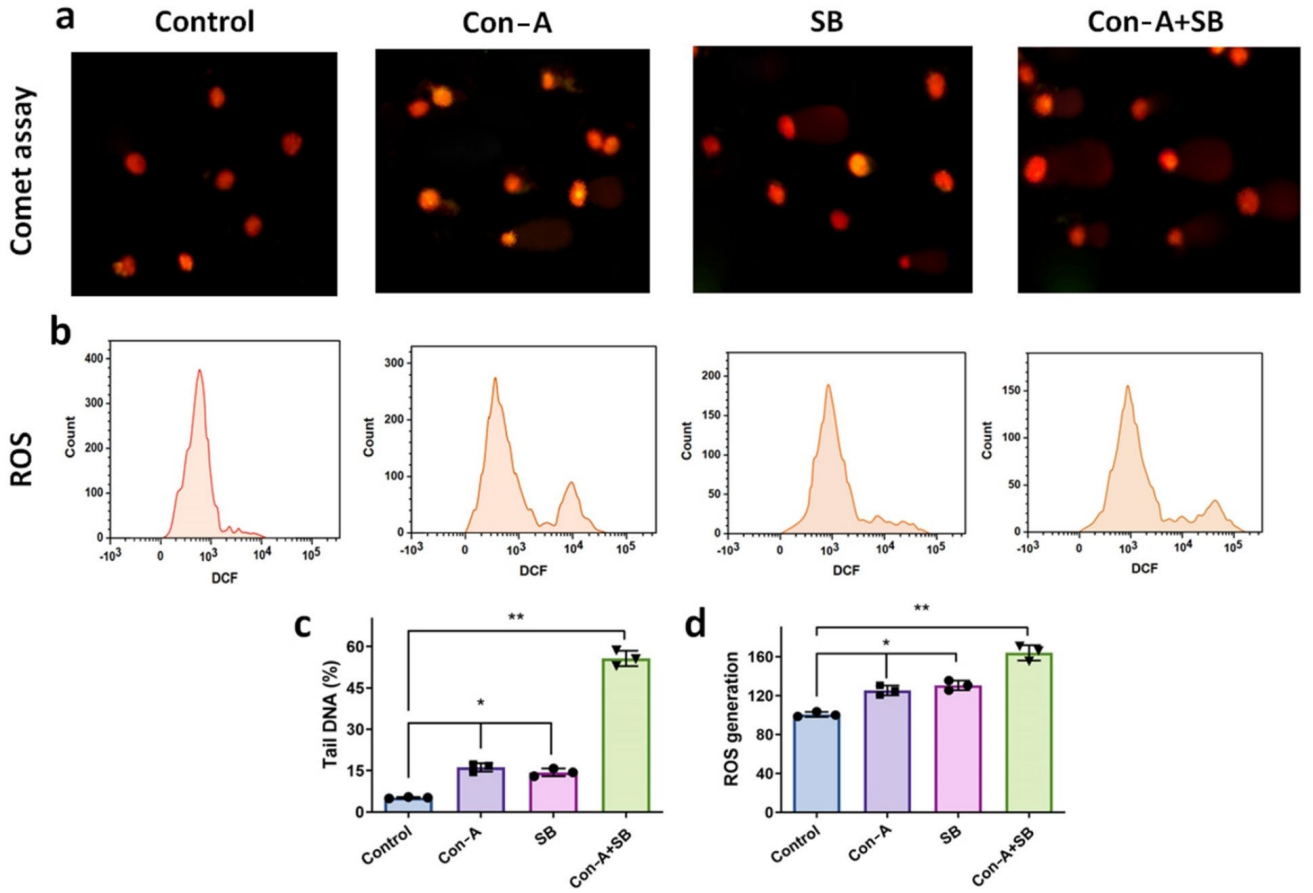
Impact of Con-A, SB, and Con-A + SB combo on AGS cell DNA damage and intracellular level of ROS generation. **a** AGS cell lines that had been grown with Con-A, SB and combination of Con-A and SB were used to conduct the Comet assay. The calculations for head, tail instance, and tail length were performed using CASP software. Despite this, when they were combined, the degree of DNA damage elevated. It has been determined that the combined use of Con-A and SB can induce DNA breakage in gastric cancer cells at concentration associated manner, a sign of death of cells by apoptosis. **b** The investigation measured the intracellular level of ROS generation in gastric cancer cells employing a DCFH-DA probe, and flow cytometry and confocal imaging were used to confirm the results (IC_50_ concentration). **c** Bar graph showing the proportion of tail DNA assessed with Comet Assay Software Programming. **d** Comprehensive evaluation of ROS levels inside cells. The aforementioned results are all the mean ± standard deviation of three separate studies, with ∗ *P* < 0.05 and ∗  ∗ *P* < 0.01

The comet assay was used to measure the amount of DNA breakage in the cells of gastric carcinoma (48 h) in the presence of Con-A, SB and combo with Con-A + SB at dose dependent way. Figure 3a illustrates how DNA damage caused by IC_50_ concentration of Con-A, SB was seen to be progressive in rise in matching comet tail length. Figure 3c illustrates the picture dimension in the event that notable tail lengths were recorded. Nevertheless, their IC_50_ concentration of Con-A + SB combination increased the degree of DNA damage. Based on Fig. 3, it can be concluded that Con-A + SB together had the ability to induce DNA fragmentation in gastric carcinoma cells I concentration dependent manner, which is evidence of cell death caused by apoptosis.

### Significant generation of ROS with the combined action of Con-A and SB

The current investigation measured the intracellular level of ROS generation in gastric cancer cells employing a DCFH-DA probe, and flow cytometry and confocal imaging were used to confirm the results. The flow cytometry findings showed that the simultaneous administration of Con-A + SB significantly enhanced generation of ROS (Fig. 3b) in contrast to the untreated group after treatment with Con-A, SB and Con-A + SB for 24 h. Using flow cytometry, the magnitude of DCF fluorescence was determined (Fig. 3d), and the levels of ROS in the cells were quantitatively assessed. AGS cells produced more ROS after receiving concurrent treatment with Con-A + SB, and this increase was dose-dependent. These studies elucidated the intrinsic apoptotic signaling mechanisms of gastric cancer cells.

### Apoptosis determination

**Fig. 4:**
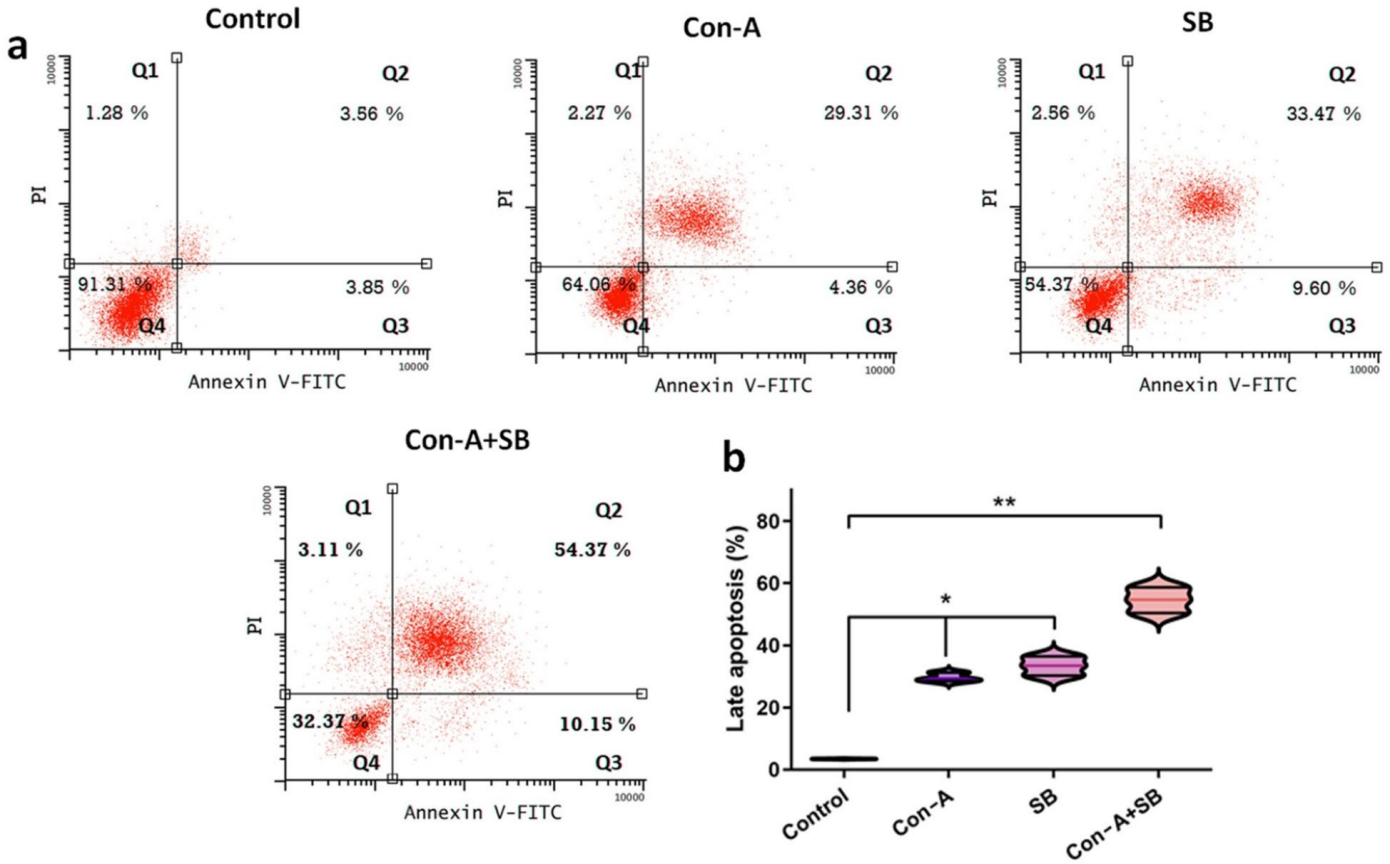
Con-A and SB together dramatically increased the rate at which AGS cells underwent apoptosis. IC_50_ concentrations of Con-A, SB and combination of Con-A + SB were applied to AGS cells for duration of 24 h. Next, flow cytometry was used to assess the progression of apoptosis. **A** Induction of apoptosis in AGS cells by Con-A along with SB. **B** Analysis of the data in (**A**). The values are shown as the mean ± standard deviation of three assessments. Significant differences between groups are indicated by ^∗^*P* < 0.05 and.^∗∗^*P* < 0.01

Annexin-V and PI double labeling were used to determine if the combined use of Con-A + SB caused cell apoptosis in AGS cells. The apoptotic percentages for the control group were 3.56% ± 1.2%. Significant differences were seen between the AGS cells treated with each combination of Con-A + SB and the untreated control group. The comparable percentages of apoptotic cells were 29.3% ± 1.1%, 33.47% ± 1.18%, and 54.37% ± 2.12% Con-A, SB and Con-A + SB (Fig. 4a-b). While cells received therapy with Con-A + SB, there was a significant rise in cell apoptosis.

### Examining the cell cycle with flow cytometry

**Fig. 5:**
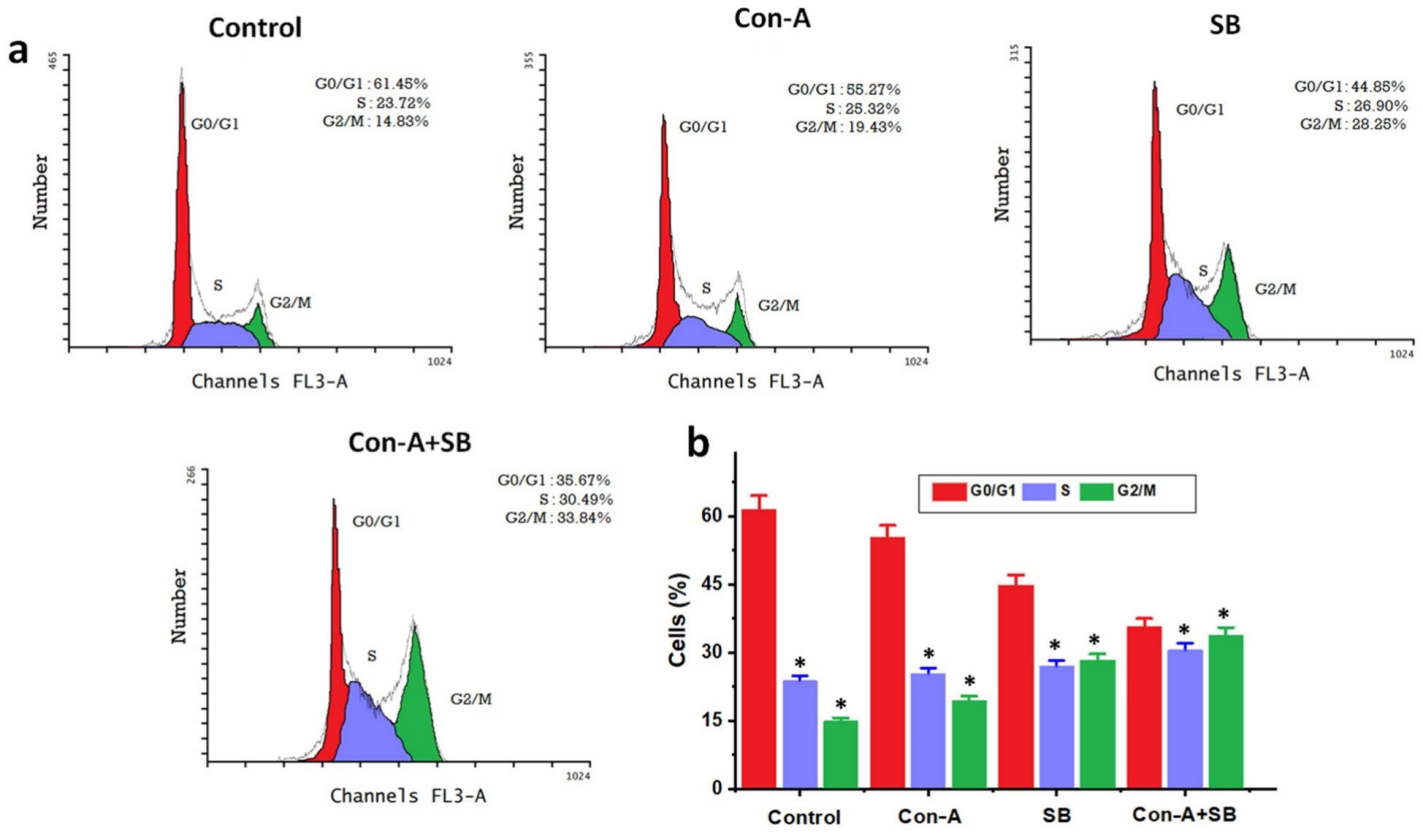
**C**ombined impact of SB and Con-A on the pattern of cell cycles in human gastric cancer cells. The IC_50_ doses of Con-A, SB and Con-A + SB combined were applied to AGS cells for a duration of 24 h. Following the completion of these treatments, adherent and non-adherent cells were separated and kept at 4˚C for an overnight incubation with 70% ethanol. **a** AGS cells’ DNA composition was examined using a flow cytometer. From this results the combination of Con-A + SB treatment for AGS cells produced an arrest in the G2/M phase of the cell (**b**) The distribution of cell cycles in and the percentages of cells shown. The values are shown as the mean ± standard deviation of three assessments. Significant differences between groups are indicated by ^∗^*P* < 0.05

Controlling the growth of cancer cells requires the cell cycle to be advanced. To learn more about the potential mechanism causing the combined use of Con-A + SB's anti-proliferative capabilities, the effects of Con-A, SB and Con-A + SB on cell cycle distribution were examined. Con-A + SB co-treatment at IC_50_ concentrations for AGS cells produced an arrest in the G2/M phase of the cell, according to the results of cell cycle tests. This suggests that the combined administration of Con-A + SB inhibited cell growth by terminating the cell cycle at dose dependent way (Fig. 5a-b).

### Determination of mitochondrial membrane potential (Δψm)

**Fig. 6:**
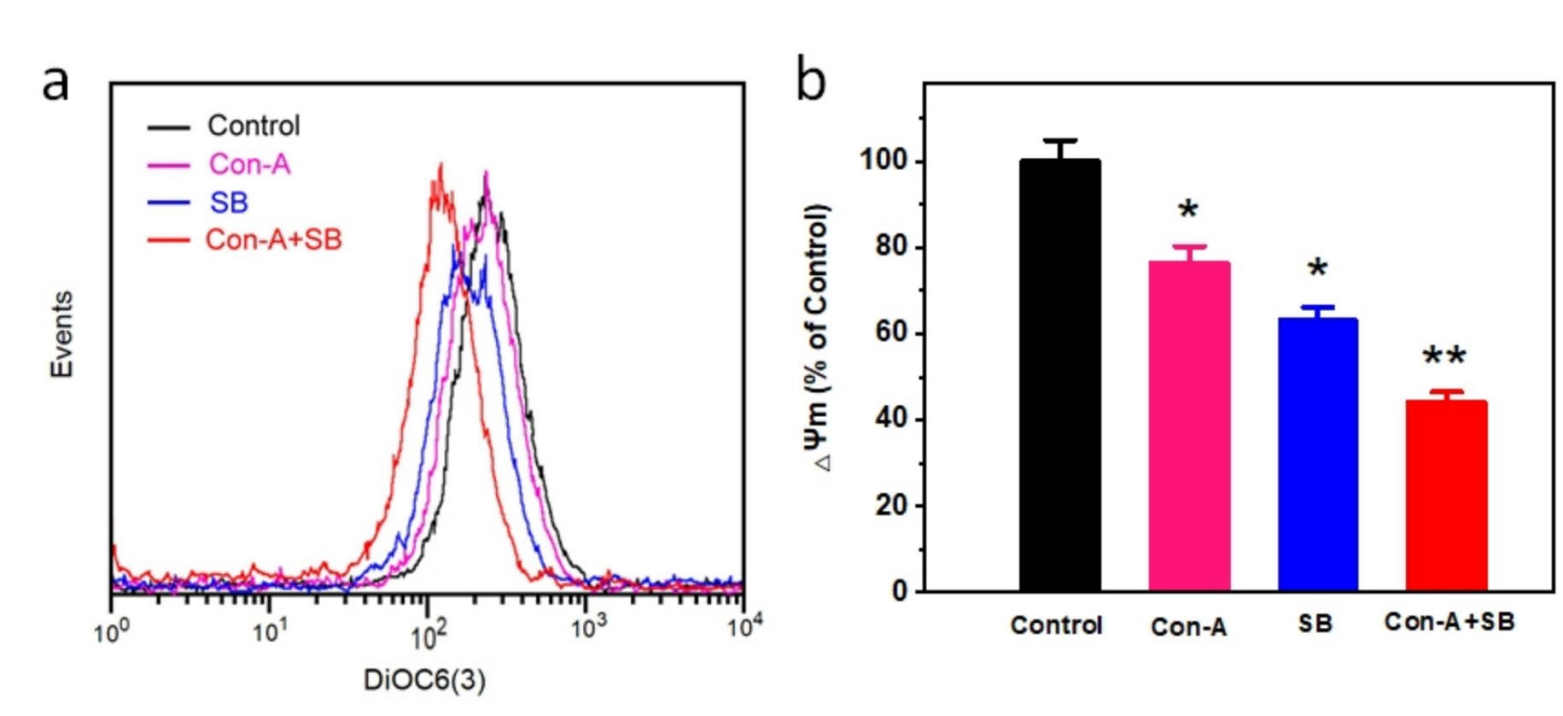
The combined effect of Con-A and SB on MMP levels in AGS cells. After 24 h of exposure to IC_50_ dosages of Con-A, SB, and the Con-A + SB combination, cells were treated with DiOC6(3) and flow cytometry was used to quantify them. **A** AGS cells demonstrated ΔΨm implosion. **B** ΔΨm levels were represented as a percentage of MFI relative to the control group. Data are presented as the mean ± SD of at least three assessments. *p < 0.05 and **p < 0.01 indicate significant variation between groups

To assess mitochondrial integrity, AGS cells were administered with IC₅₀ doses of Con-A (19.6 μM), SB (16.78 μM), and the combination Con-A + SB (10.37 μM) for 24 h. Figure 6 shows that Con-A-treated cells retained 76.54 ± 2.07% of ΔΨm compared to control cells, showing a significant decline. SB therapy significantly reduced ΔΨm to 63.12 ± 3.21%, suggesting early mitochondrial impairment. The combination treatment (Con-A + SB) significantly reduced ΔΨm to 44.24 ± 2.11% (*p* < 0.01), indicating mitochondrial membrane depolarization. These findings show that Con-A + SB increases mitochondrial dysfunction more efficiently than either drug alone. This finding suggested that the alteration of mitochondrial membrane potential by Con-A + SB causes apoptosis based on the concentrations. Additionally, the combination's effect outperformed Con-A, SB alone, which was in line with the recognition of apoptosis.

### Binding interaction of Con-A and SB with JAK/STAT3

**Fig. 7:**
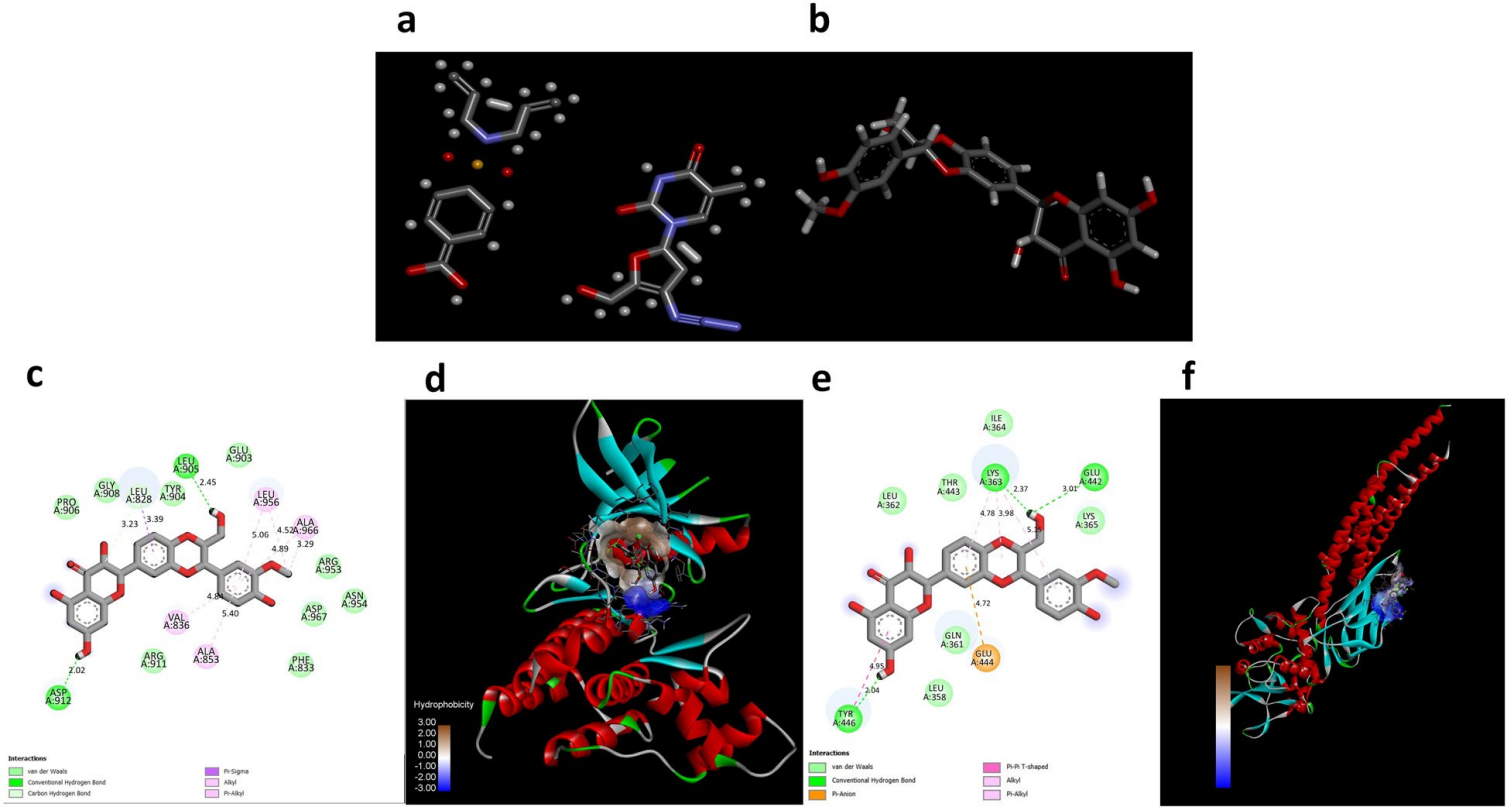
The two- and three-dimensional interactions between bioactive compounds and the key target protein JAK/STAT3 were analyzed. Specifically, (**a**, **b**) chemical structure of Con-A and SB. **c**, **d** two- and three-dimensional interactions of bioactive compounds with target protein JAK and (**e**, **f**) two- and three-dimensional interactions of bioactive compounds with target protein STAT3. These interactions were visualized using Biovia Discovery Studio, which produced both 2D and 3D structural representations

By precisely estimating a ligand's form inside the boundaries of an attachment pocket, docking aims to evaluate the strength of attachment appropriately. To determine their potential mechanism of action, the bioactive compounds Con-A and SB were docked with specific proteins JAK and STAT3 to assess their affinity with their binding sites. Among the top-ranking targets (JAK/STAT3), the JAK revealed the highest binding affinity with SB at – 6.46 kcal/mol, followed by STAT at – 5.70 kcal/mol (Fig. 7).

### Con-A + SB combine inhibits JAK/STAT3 in AGS cell

**Fig. 8:**
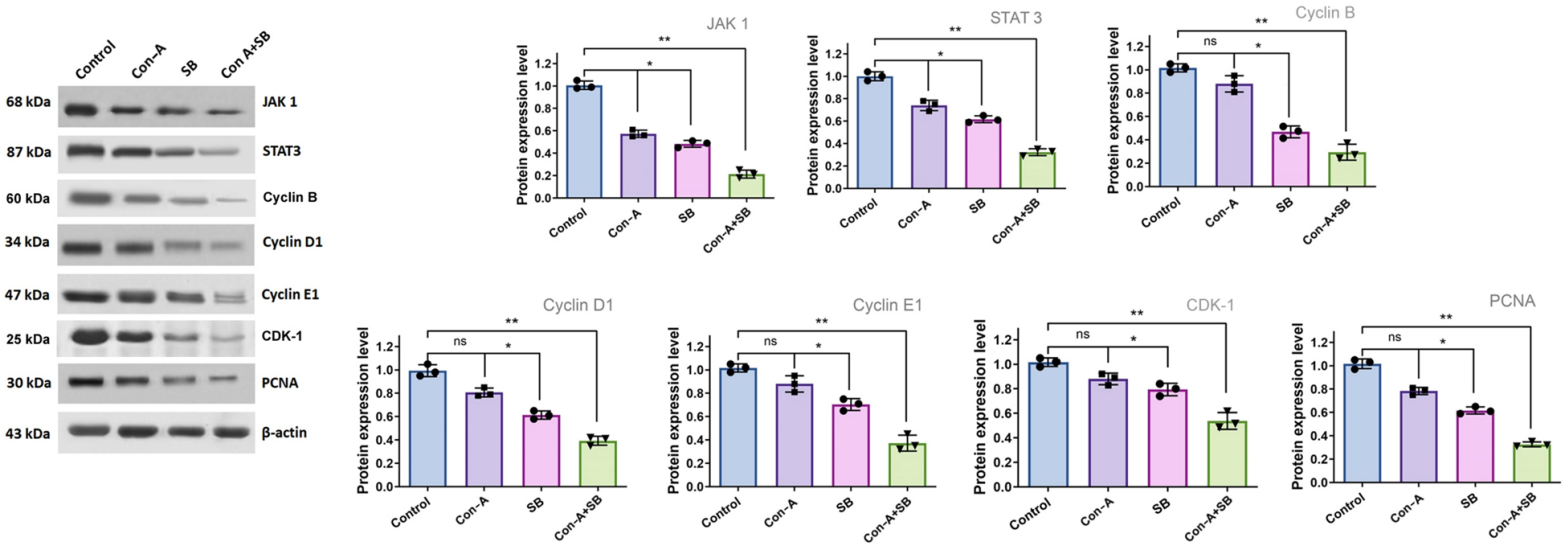
In gastric cancer cells, the combined use of SB and Con-A caused the suppression of JAK2/STAT-3 phosphorylation. Three different trials’ worth of protein expression was standardised to β-Actin. Densitometric measurement was done using Image J software. The values are shown as the mean ± standard deviation of three assessments. Significant differences between groups are indicated by *p < 0.05 and **p < 0.01

**Fig. 9:**
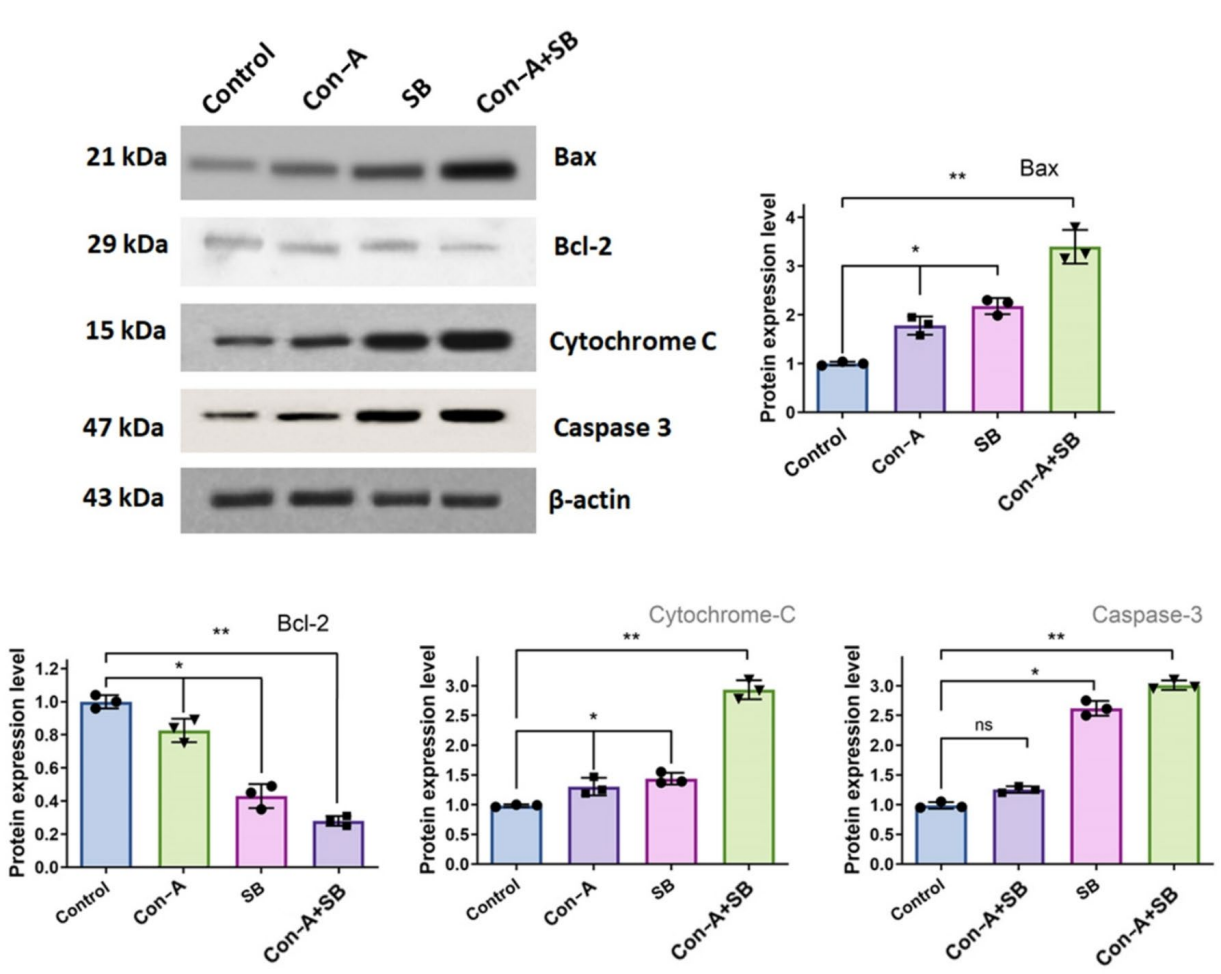
The expression of pro- and anti-apoptotic proteins in AGS cells was found to be caused by Con-A, SB and combination of Con-A + SB, as determined by western blot analysis. Pro-apoptotic Bax, Cytochrome C, caspases-3 and −9 are definitely expressed more than anti-apoptotic proteins like Bcl-2 protein expression, as the figure clearly illustrates. To quantify densitometrically, Image J program was utilized. Each of the three experiments'protein expression levels was adjusted to β-actin. The values are shown as the mean ± standard deviation of three assessments. Significant differences between groups are indicated by *p < 0.05 and *p* ** < 0.01

In the present work, we have identified the cellular mechanisms below the loss in cell growth by examining the proteins that are required for these natural events. Western blot analysis was used to identify the protein that triggers apoptosis in gastric cancer cells that were assessed using IC_50_ doses of Con-A, SB and Con-A + SB. We examined the expression levels of cyclin B, Cdk1, cyclin D1, PCNA, JAK1, STAT3, and other cancer pathway components (Fig. 8). Based on these data, we inferred that Con-A + SB-treated cells had significantly lower expression levels of proteins linked to tumor formation, such as JAK1, STAT3, PCNA, cyclin D1, along with evidence of G2/M phase arrest, as indicated by the downregulation of Cyclin B and Cdk1. It has been demonstrated, nevertheless, that the expression of control cells was elevated. Furthermore, Con-A + SB induces apoptosis in human gastric cancerous cells, resulting in the induction of caspase 3 and Bax, alongside a discharge of cytochrome C through the mitochondria and a decline in Bcl-2 expression (Fig. 9). These findings supported our preexisting knowledge that Con-A + SB suppresses the JAK/STAT3 signaling cascade in AGS cells and the activity of cell cycle-regulating proteins. Furthermore, in gastric cancer, the combination treatment's impact on inhibiting the JAK/STAT3 pathway was greater compared to the use of separate treatments.

## Discussion

Effective treatment of cancer is difficult due to its complexity, involving several pathways and chemicals. Repurposed drugs and combination treatments, among other therapeutic methods, are being extensively researched in the ongoing research for this disease, which includes therapy for gastric cancer. Combining drugs is a common strategy used to enhance cancer treatment, lowering its toxicological impact and raising its effectiveness. But there continue to be drawbacks, mostly financial and toxicological ones, despite the increased research and interest in these approaches [27]. According to recent studies conducted in vivo and in vitro, phytochemicals have been shown to have chemopreventive effects in a variety of tumor models [28]. Concanavalin A and silibinin together generate a synergistic form that inhibits cell growth in AGS gastric tumor cells, a finding that is consistent with the current work.

The major active ingredient in silymarin, silibinin is a flavonolignan that is discovered in milk thistle seeds. This compound is an increasingly common nutritional supplement that has been extensively studied for its anti-oxidants, liver-protective, and cancer prevention properties [29]. In many pre-clinical studies malignancy models, such as those for prostate, colorectal, lung, and breast carcinoma, it has also demonstrated a strong degree of efficacy in suppressing or postponing the development of tumors as well as their propagation occurring [22]. According to Lu et al. [30], silibinin's cancer-fighting properties are linked to a number of signaling mechanisms that are connected to cell growth. Cecen et al. [31], have reported that silibinin exhibits protective benefits against doxorubicin-induced toxic relationships, which makes it substantially more appealing.

Many different organisms contain lectins, such as Con-A, which are glycoproteins. The majority of these lectins are extracted from plant materials [32]. Con-A has been shown in earlier research to trigger apoptosis in mouse macrophage PU5-1.8 cells and human melanoma A375 cells. Furthermore, in HeLa cells, Con-A caused autophagic cell death [19, 33]. Consequently, Con-A has strong apoptosis- and autophagy-inducing qualities, which could potentially render it an effective anti-neoplastic drug for upcoming cancer treatments. Additionally, they possess a high degree of selectivity in their weak binding to glycans, which allows them to form glycoconjugates [34]. Con-A has really been effectively utilized in the creation of anticancer drug-loaded nanoparticles that have the ability to become internalized and preferentially adhere to cancer cells that excessively express membrane glycans [35]. Con-A treatment of gastric cancer cells is still unreported.

The purpose of this investigation is to determine whether or not the combination of Con-A + SB functions against the AGS cells and to look into potential processes as well as the impacts of this combination. The cytotoxicity effect of Con-A + SB in AGS cancer cell lines, the exact mechanism of action of these flavonoids remains mostly unknown. We assessed the individual and combined cytotoxicity of Con-A + SB in our investigation. Combinations of Con-A + SB were added to cells at escalating concentrations, and their cytotoxic impact was measured using the MTT assay. The AGS cell line exhibited dose-dependent cytotoxicity upon treatment with Con-A and SB, with calculated IC₅₀ values of 19.6 ± 1.3 μM and 16.78 ± 1.1 μM, respectively (*n* = 3, *p* < 0.05). Thus, our findings demonstrated that, when administered to the AGS gastric cancer cell line, Con-A and SB together produced noticeably greater toxicity than when administered separately.

Apoptosis is a type of planned cell death that involves several different molecular pathways and is essential to the development and upkeep of cells and their machinery seen in healthy tissues. The past study has shown that cancer can result from insufficient apoptosis [36, 37]. The DNA damage checkpoint system, which is triggered by a number of illnesses, can minimize harmful DNA-damaged cells by causing apoptosis, which prevents cancer. As a result, apoptosis preserves genomic integrity, yet imbalanced processes related to apoptosis may potentially promote carcinogenesis and result in cancers that are resistant to therapy. Hence, one of the characteristics of cancer is the evasion of apoptosis [38–41].

The features of apoptosis, such as nuclear alterations and apoptotic nucleus development, are studied by fluorescence microscopy analysis of DAPI-stained cells. In AGS gastric cancer cells, cells treated with synergy with Con-A + SB displayed apoptotic characteristics. Apoptotic cell growth, chromatin condensate to and intracellular and subcellular reduction are the signatures of apoptosis. Administration with IC_50_ concentration of Con-A, SB and combined use of Con-A + SB elicited morphological alterations far greater than their control group effects. A characteristic feature of the apoptotic process is the creation of nuclear DNA fragmentation. DNA fragmentation was previously used to demonstrate that Rutin and Silibinin cause’s apoptotic cell death in earlier research [42]. Therefore, in order to ascertain the potential mechanism of cell death induced by 5, 10, 15 µM combined effect of Con-A + SB in gastric cancer cells, the DNA gel electrophoresis approach was employed. Through an apoptotic process, the cytotoxic impact of their combined treatment enables the formation of apoptotic DNA fragments more frequently in the combo compared to their independently therapies on agarose gel.

Comet test is employed in the observation of DNA breakage in individual cells resulting from exposure to genotoxic substances, such as pesticides, dioxins, chemotherapy, and radiotherapy in cancer patients, as well as in the assessment of chemopreventive drugs'genoprotective qualities. It works with both cancerous and normal cells, allowing for the identification of each single- and double-strand break. An earlier investigation utilizing the Comet Assay in colon cancer cells showed that silibinin enhanced DNA damage [42]. DNA damage in gastric cancer cells was examined in our work utilizing the comet assay in with the addition of Con-A, SB and Con-A + SB combination. A steady rise in the equivalent comet tail length indicated that Con-A + SB combination had caused DNA damage. Nevertheless, the combined effect of Con-A + SB increased the degree of DNA destruction. It is now known that the combination of Con-A and SB caused DNA breaks in gastric cancer cells.

The mitochondrial membrane on the inner surface undergoes modifications due to apoptosis, chemotherapeutic medicines, or other external factors. This results in the loss of the mitochondrial membrane potential (ΔΨm) and the expulsion of cytochrome c throughout the mitochondria towards the cytosol. Biochemical indicators of apoptosis via the mitochondrial route include the collapse of ΔΨm and the release of cytochrome C [25]. In this work, Con-A induced a significant drop in ΔΨm, whereas SB dramatically altered mitochondrial potential. The combination of Con-A and SB significantly diminished ΔΨm, representing improved mitochondrial dysfunction. This synergistic effect shows that the combo treatment is more effective at inducing mitochondrial-mediated apoptosis. The increased depolarisation found in the Con-A + SB group indicates the participation of mitochondrial pathways in the mechanism of action, which may explain the combination treatment's stronger apoptotic efficacy than either agent alone. Similar changes in ΔΨm have been documented in breast and colon cancer models, indicating mitochondrial dysfunction as a crucial characteristic for apoptosis induction [43, 44]. Furthermore, the combination's effect outperformed that of control, which was in line with the detection of apoptosis. Furthermore, the production of ROS in tumor cells represents one of the processes by which IC_50_ dosages of Con-A, SB and Con-A and SB combination exhibit their anticancer effects. The level of intracellular reactive oxygen species might be estimated using the fluorescence magnitude of DCF. The results of the study showed that both the qualitative as well as quantitative assessment of ROS, the fluorescence intensity of the combo of Con-A + SB administered to cells was significantly greater compared to the treatment of the control group.

Addressing the cell cycle in addition to cell death has progressed chemotherapy for centuries. Unregulated cell growth would result from a dysregulated cell cycle, which would also allow cells to multiply and proliferate unrestrictedly [45]. Targeting the cell cycle with reduced cell division may thereby halt the development of cancer [46]. Cyclins are essential for initiating and expediting the cell cycle transition from the G1 to the S and G2/M phases. It has been suggested that cyclin D1 may have a role in different cycle states. Cyclin D1, also known as G1/S-specific cyclin-D1, is generally involved in promoting cell proliferation through the combination and activation of the cycle dependency protein kinase CDK4's G1 phase characteristic [47]. However, our analysis revealed a higher drop in the G2/M cell cycle. To further evaluate the potential cell cycle arrest caused by Con A, SB, and their combination, the expression of G2/M phase-specific markers Cyclin B and Cdk1 was measured by Western blot analysis. Notably, the combined treatment (Con-A + SB) resulted in a substantial decrease in the expression of each marker, including cyclin B and cdk1, revealing a synergistic impact that accelerates G2/M phase arrest. These findings suggest that, in addition to G1 arrest mediated via Cyclin D1 and E1 downregulation, the Con A and SB combination also impacts the G2/M transition, potentially enhancing the antiproliferative effects on cancer cells. This suggests that Con-A and SB may influence distinct signaling pathways to control cyclin D1, expression, where cyclin D1 acts as a downstream regulator of cell survival [48]. It has been suggested that Con-A and SB may partially inhibit cyclin D1 activity. In contrast to the categories for which medicines were used independently the combination therapy of these flavonoids led to a significant drop in the quantity of intracellular cyclin D1.

Component in numerous cancers and cell lines, STAT3 activation plays a crucial part in the formation and carcinogenesis of malignancies. Through the control of numerous downward target genes, STAT3 may boost angiogenesis, support cancer cell growth and spread, suppress apoptosis, and encourage cellular growth and survivability [49]. According to multiple findings, triggers of STAT3 may be utilized as a molecular grading indicator for determining a bad prognosis for gastric carcinoma and related to the disease's growth and invasiveness. Prior research has demonstrated that inhibiting activated STAT3 can stop chemotherapy-induced resistance within human gastric carcinoma cells [50, 51], and that focusing on stomach epithelial STAT3 specifically may be therapeutically efficient at hindering the development of gastric cancer [52]. Thus, GC treatment may be able to effectively target the suppression of the elevated STAT3 signaling cascade. When Con-A and SB were combined, there was a discernible down-regulation in the expression of the STAT3 and JAK1 proteins while compared to the control and drug alone. Apoptosis in cells of gastric carcinoma may be induced primarily via these proteins.Through western-blot analysis, we were able to determine the fact that in gastric cancer cells, IC_50_ concentration of combined use of Con-A and SB all expressed more apoptotic circuit proteins than did each of the therapies alone. Anti-apoptotic protein Bcl-2 was shown to be downregulated, whereas pro-apoptotic protein Bax was found to be expressed more frequently concurrently. In addition, there was an increasing the activity of caspase 3 and 9 and an induction of the mitochondrial intrinsic route, which led to apoptosis. Thus, our work demonstrated that the combined use of Con-A and SB caused AGS cells to undergo apoptosis via inhibiting the JAK/STAT3 signaling pathway, in addition to simultaneously up-regulating caspases and down-regulating Bcl-2.

The increasing focus in cancer immunotherapy has focused attention on the JAK/STAT3 signaling system, which regulates not only tumor development additionally immune evasion and therapeutic response. The current clinical research found that combining oxaliplatin with Teysuno (SOX) with immune checkpoint inhibitors for the transformation treatment of locally advanced gastric cancer resulted in better patient survival results [53]. Similar combinatorial methods are consistent with our results on the synergistic impact of Concanavalin A and Silibinin, both of which not only display cytotoxic and pro-apoptotic activities, but additionally inhibit STAT3 signaling, a critical modulator of immune suppression and tumor progression. These findings imply that Con-A and SB may help to boost immunological sensitivity in gastric cancer.

Additionally, emerging biomedical developments, including CRISPR-based gene control, AI-driven drugs repurposing, organ-on-a-chip models, and nanotechnology-assisted system of delivery are speeding up translational investigations into cancer [54]. The use of these techniques can assist in customize therapy and improve cancer prognosis. Our molecular docking study enables the use of in silico techniques to find and optimize natural chemical interactions with oncogenic targets such as JAK/STAT3, establishing the framework for rational drug design and combination treatment tactics. Overall, this study merely supports the new direction of phytochemicals, notably Concanavalin A and Silibinin, in the treatment of gastric cancer, but it also coincides with current biomedical innovation trends targeted at personalizing and improving cancer care.

The JAK/STAT3 pathway is critical to cancer cell survival, proliferation, immune evasion, and treatment resistance [55]. Innovative methods, which include PROTACs (Proteolysis Targeting Chimeras), which allow for the selective breaking down of oncogenic proteins, and siRNA-based therapies, are being investigated to target STAT3 at both the transcriptional and post-translational stages. Furthermore, nanoparticle-based drug delivery technologies provide tailored STAT3 regulation in the tumour microenvironment, reducing systemic toxicity and increasing therapeutic effectiveness [56].

Recent advances in liquid biopsy technology have created new opportunities for non-invasive cancer diagnostics, including real-time monitoring of therapy response and recurrence of the tumor. For example, in neuroblastoma, circulating tumor DNA (ctDNA) is useful for monitoring therapy results and detecting relapse episodes [57]. Similarly, molecular barcoding techniques have enhanced the sensitivity and specificity of mutation detection in pancreaticobiliary tumors, allowing for more precise diagnoses in clinical oncology [58]. Furthermore, DNA methylation profiles have emerged as accurate biomarkers for early-stage cancer diagnosis, particularly in breast cancer, opening up new avenues for screening and disease differentiation [59].

Given our findings that Con-A and SB combined therapy regulates apoptosis, interferes with mitochondrial membrane potential, increases ROS levels, and inhibits STAT3/JAK1 signaling, it is possible that liquid biopsy techniques could be used to track biomarkers expressing these molecular processes, that include modified methylation of apoptotic genes or expression signatures in ctDNA. Future research could investigate if the therapeutic response to Con-A and SB in gastric cancer can be evaluated using such non-invasive biomarker platforms, adding translational relevance to our mechanistic findings.

## Conclusion

Chemotherapeutic agents for cancer are largely directed towards fast-growing tumour cells and can be found in abundance in natural products. The results of this study demonstrated that in human gastric cancer AGS cells, the combination of Con-A and SB decreased proliferation, promoted apoptosis, and triggered cell cycle arrest at the G2/M phase. A decline in STAT3, down-regulation of Bcl-2 in conjunction with enhanced regulation of caspase 3 and caspase 9, and a reduction in the cell-cycle controlling component cyclin D1 are among the molecular processes linked to Con-A and SB effectiveness. When combined, Con-A and SB may inhibit malignancy in AGS cells through the JAK/STAT3 pathway. Our findings highlight the synergistic anticancer potential of SB and Con-A, emphasising their ability to selectively target tumour cells and enhance chemotherapeutic efficacy.

## Supplementary Information

Supplementary Material 1.

## Data Availability

No datasets were generated or analysed during the current study.
