# Supplementary figures and images for: Synergistic inhibition of gastric cancer cell proliferation by concanavalin A and silibinin via attenuation of the JAK/STAT3 signaling pathway and molecular docking analysis
Source: Hereditas. 2025 May 11;162:73. doi: 10.1186/s41065-025-00438-z (PMC12067676; doi:10.1186/s41065-025-00438-z)

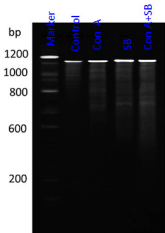

Supplement: Supplementary file 1 — Supplementary Material 1. [file 41065_2025_438_MOESM1_ESM.zip › Fig 2b-Uncropped Gel.pdf]

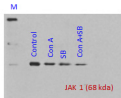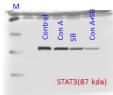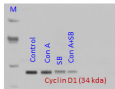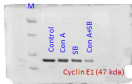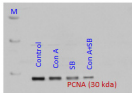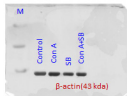

Supplement: Supplementary file 1 — Supplementary Material 1. [file 41065_2025_438_MOESM1_ESM.zip › Fig 8-Uncropped Blots.pdf]

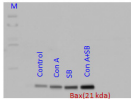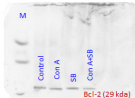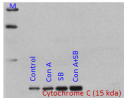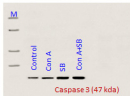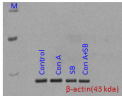

Supplement: Supplementary file 1 — Supplementary Material 1. [file 41065_2025_438_MOESM1_ESM.zip › Fig 9-Uncropped Blots.pdf]
